# Medial-lateral translational malalignment of the prosthesis on tibial stress distribution in total knee arthroplasty: A finite element analysis

**DOI:** 10.3389/fbioe.2023.1119204

**Published:** 2023-03-02

**Authors:** Zhiqian Zheng, Yang Liu, Aobo Zhang, Hao Chen, Qian Wan, Lei Zhong, Xiaonan Wang, Qing Han, Jincheng Wang

**Affiliations:** Department of Orthopedics, The Second Hospital of Jilin University, Changchun, China

**Keywords:** total knee arthroplasty, malalignment, finite element analysis, tibial stress, wear rates

## Abstract

**Background:** Poor prosthesis alignment during total knee arthroplasty could cause problems such as polyethylene spacer wear, leading to surgical failure and revision surgery. The problems caused by the malalignment of the tibial plateau prosthesis in the medial and lateral planes are unclear. We aimed to investigate the stress distribution and micromotion of the tibia when the tibial plateau prosthesis is translated 1 and 2 mm medially and laterally, respectively, using finite element analysis (FEA).

**Method:** A non-homogeneous tibia model was created and load conditions when standing on two legs were applied using FEA to simulate the misaligned prosthesis. The stresses, stress distribution, and micromotion of the proximal tibia were analyzed in five positions of the tibial plateau prosthesis: Lateral-2 mm; Lateral-1 mm; Medium; Medial-2 mm; Medial-1 mm.

**Result:** The maximum stress in the five groups with different misalignments of the platform was 47.29 MPa (Lateral-2 mm). The maximum micromotion among the five groups in different positions was 7.215 μm (Lateral-2 mm).

**Conclusion:** When placing the tibial plateau prosthesis during total knee arthroplasty, an error of 2 mm or less is acceptable as long as it does not overhang.

## 1 Introduction

Several studies have shown that total knee arthroplasty (TKA) is an effective and durable treatment for end-stage knee arthritis (Vessely et al., 2006; Guo et al., 2012). The primary goals of TKA include reducing knee pain, re-aligning the femur and tibia, maintaining knee stability, and preserving joint flexibility. Despite the great clinical results of TKA, revision rates remain high due to poorly aligned prosthetic components, resulting in aseptic loosening, instability, and polyethylene wear (Dalury et al., 2013).

To predict and avoid the problems mentioned above and thus improve the prognosis of surgery, the analytical approach (Jin et al., 1995), experimental measurement (Liau et al., 1999) and finite element analysis (Popescu et al., 2019; Dong et al., 2020; Park et al., 2021) (FEA) have been widely used in the field of orthopedics. Matsuda et al. (1999) investigated the effect of varus tilt on contact stresses in total knee prostheses using electronic pressure sensors; Liau et al. (1999) studied the effect of tibiofemoral joint contact alignment of knee prosthesis using Fuji pressure-sensitive film in an *in vitro* biomechanical test. They also explored the effect of the tibial polyethylene component of the total knee prosthesis on stresses using FEA (Liau et al., 2002). Different misalignment conditions are tested by FEA by simulating angles, friction, and stresses. These findings are applied in preoperative planning to prevent potential TKA failure (Perillo-Marcone and Taylor, 2007; Minh et al., 2013). To our knowledge, there have been few studies examining the effect of medial-lateral translational misalignment of the tibial plateau prosthesis on the tibia.

Inoue et al. (2016) showed that the risk of medial tibial condylar fractures increases with increased tilt of the valgus angle of the tibial prosthesis. If the tibial prosthesis is poorly rotated, firstly, the riser of the polyethylene prosthesis will be worn further and, since the femoral and tibial prostheses are no longer matched in the same position, the soft tissue will be twisted during flexion and extension activities, resulting in a stiff knee (Bedard et al., 2011). Secondly, internal rotation can bring about lateral patellar subluxation and wear of the lateral patellofemoral joint, while external rotation can cause inward patellar trajectory, internal tibial rotation, or a change in gait (Perillo-Marcone et al., 2000; Ishii et al., 2015). Any errors that cause asymmetric loading of the joint, such as misalignment or instability of the prosthesis, could lead to increased wear rates, resulting in surgical failure and revision (Fraser et al., 2015).

We aimed to investigate the stress distribution and micromotion of the tibia when the tibial plateau prosthesis is translated 1 mm and 2 mm medially and laterally, respectively, using FEA. This may contribute to the reduction of TKA revision rates and improved satisfaction of patients after TKA.

## 2 Materials and methods

### 2.1 Establishment of a non-homogeneous three-dimensional (3D) tibia model and surgical simulation

Computed tomography (CT) scan data were recorded from a 49-year-old male volunteer. The images were imported into Mimics (v21.0, Materialise, Leuven, Belgium), and the right tibia was rebuilt as a 3D model. This research was approved by the Ethics Committee of the Second Hospital of Jilin University and the volunteer provided informed consent.

The 3D model of the right tibia and the tibial plateau prosthesis (Ai Kang A3)were prepared as STL format files, and the simulated surgery was performed in Magics (v21.0, Materialise, Leuven, Belgium). The posterior slope was set to 5°, and the tibial model was resected in the traditional surgical fashion (Indelli et al., 2016; Maderbacher et al., 2017), by removing the tibia 6 mm below the medial tibial articular surface, perpendicular to the mechanical axis. The application of this study focused on the stress distribution during the interaction of the proximal bone with the platform prosthesis, so the distal tibia was separated from the system to reduce the calculation time.

**FIGURE 1:**
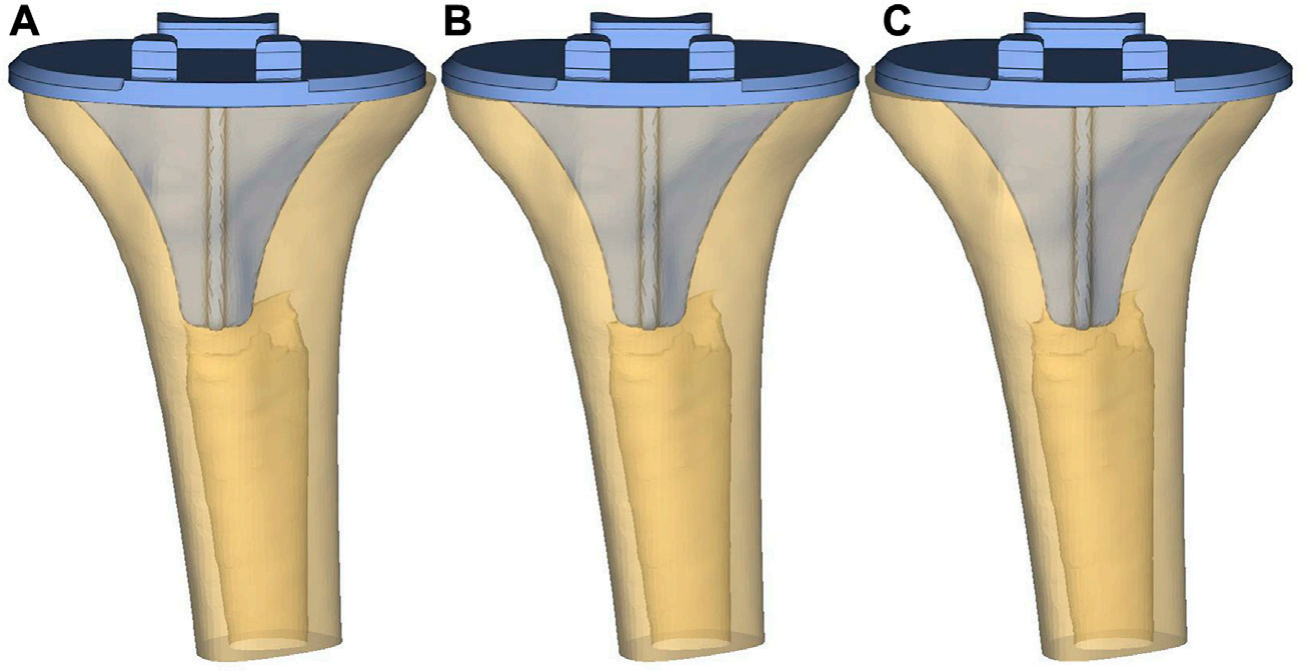
Prosthesis in different positions. **(A)** Lateral-2 mm; **(B)** Medium; **(C)** Medial-2 mm.

After installing the tibial plateau prosthesis, the prosthesis was moved medially and laterally by 1 mm and 2 mm, respectively, to simulate a slippage dislocation. The prosthesis is shown in the middle position, as well as translated 2 mm to the medial and lateral side, in Figure 1.

**FIGURE 2:**
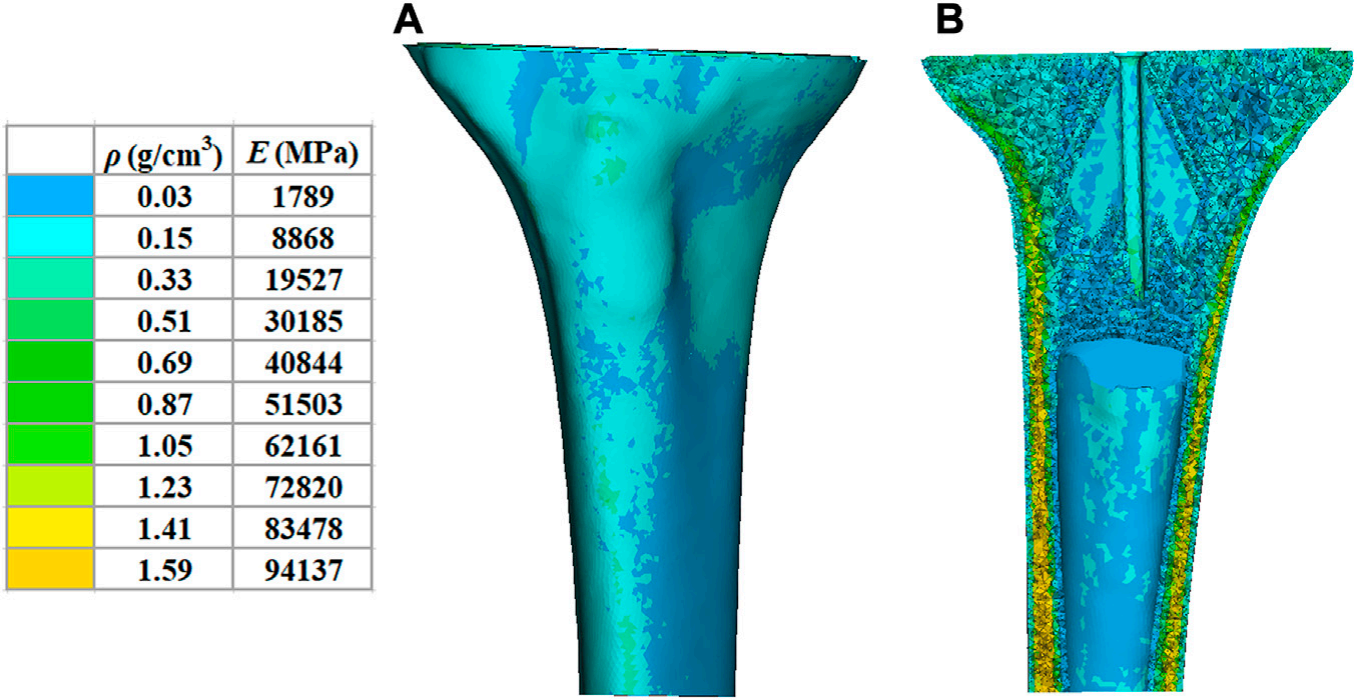
Material properties of the inhomogeneous tibia. **(A)** External material properties of the tibia. **(B)** Internal material properties of the tibia. ρ: bone density. **(E)** elastic modulus.

In the Mimics software, the 3D model of the tibia with inhomogeneous material properties was defined based on the grayscale values of the CT scans. Following previous studies, the material properties of the tibia were determined according to the following equations (Rho et al., 1995):

$$\rho \left(g/m^{3}\right)=-13.4+1017\times GV\left(HU\right)$$ (1)

$$E\left(Pa\right)=-388.8+5925\times \rho \left(g/m^{3}\right)$$ (2)

in which E is the modulus of elasticity, ρ is the bone density, and GV is the gray value of the bone in the CT data. According to other previous studies (Thompson et al., 2016), the Poisson’s ratio of the bone was set to 0.3 and the modulus of elasticity of the tibial plateau prosthesis was set to 114,500 MPa with a Poisson’s ratio of 0.3. To differentiate, the tibia was divided into ten colored regions, and the material properties are shown in Figure 2.

### 2.2 Meshing and load setting

**FIGURE 3:**
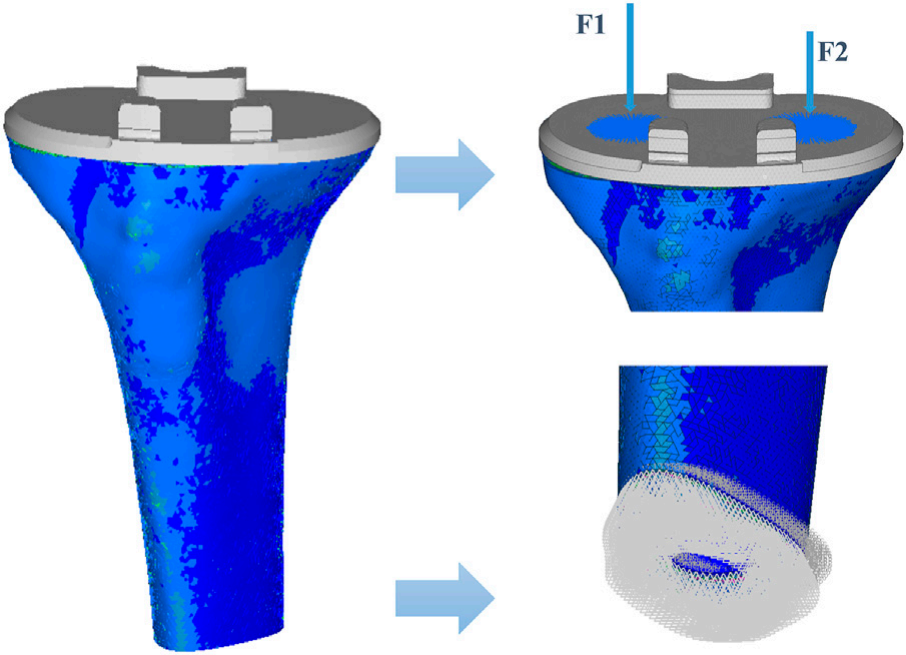
Loads and constraints of the tibia. The distal tibia is constrained completely.

All components were imported into Hypermesh (14.0, Altair, Troy, MI) to create triangular meshes with the element type C3D4. The number of elements in the bone and prosthesis are 420,036 and 117,094 respectively. A non-linear friction model with surface-surface contact was established between the superior surface of the tibia and the inferior surface of the prosthesis, and the friction coefficient was set to 0.2 (Li et al., 2019). A static analysis of the tibia was performed under a load condition of 963 N to simulate a two-legged stance (Kutzner et al., 2010). According to a previous study (Lin et al., 2017), the ratio of force between the lateral and medial tibial plateau is 40%:60%, respectively, so the force was divided into 385 N and 578 N and loaded with rigid bar element 3 (Rbe3) to transfer the force uniformly. The inferior surface of the distal tibia was constrained in all directions (Figure 3).

## 3 Results

### 3.1 Finite element analysis

#### 3.3.1 Stress

**FIGURE 4:**
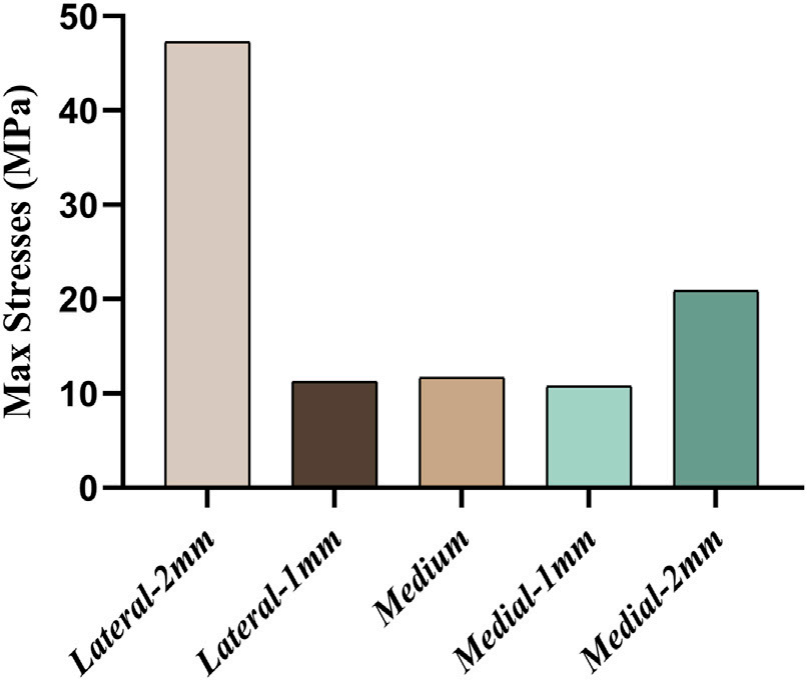
Comparison of maximum stresses when five groups of platforms are in different misalignment positions.

Figure 4 shows the maximum von Mises stress in the tibia for five sets of platforms in different positions. The highest stress peak of 47.29 MPa can be seen when the platform is misaligned by 2 mm to the lateral side, followed by the stress peak of 20.90 MPa when the platform is misaligned by 2 mm to the medial side. The remaining three data groups were not significantly different compared to each other.

**FIGURE 5:**
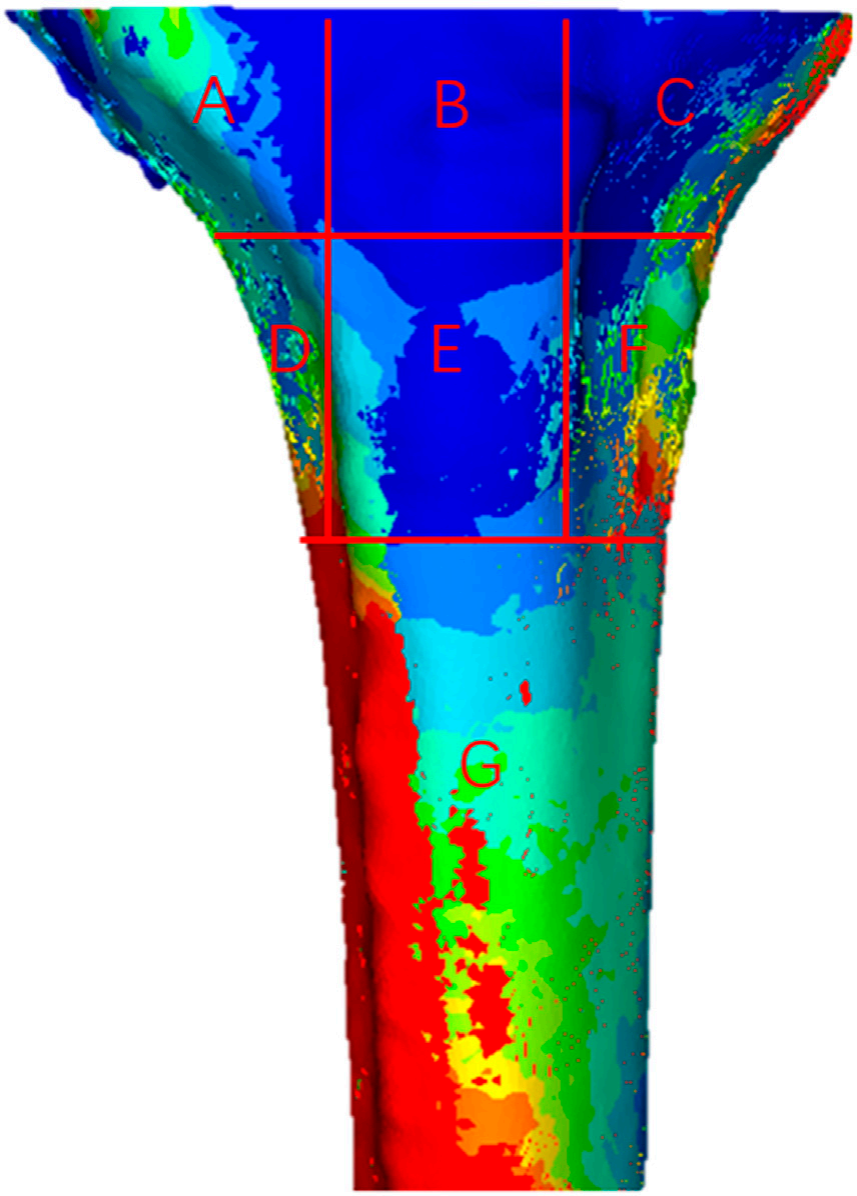
Distribution of von Mises stress in the proximal tibia. **(A–G)** Seven different regions of stress.

**FIGURE 6:**
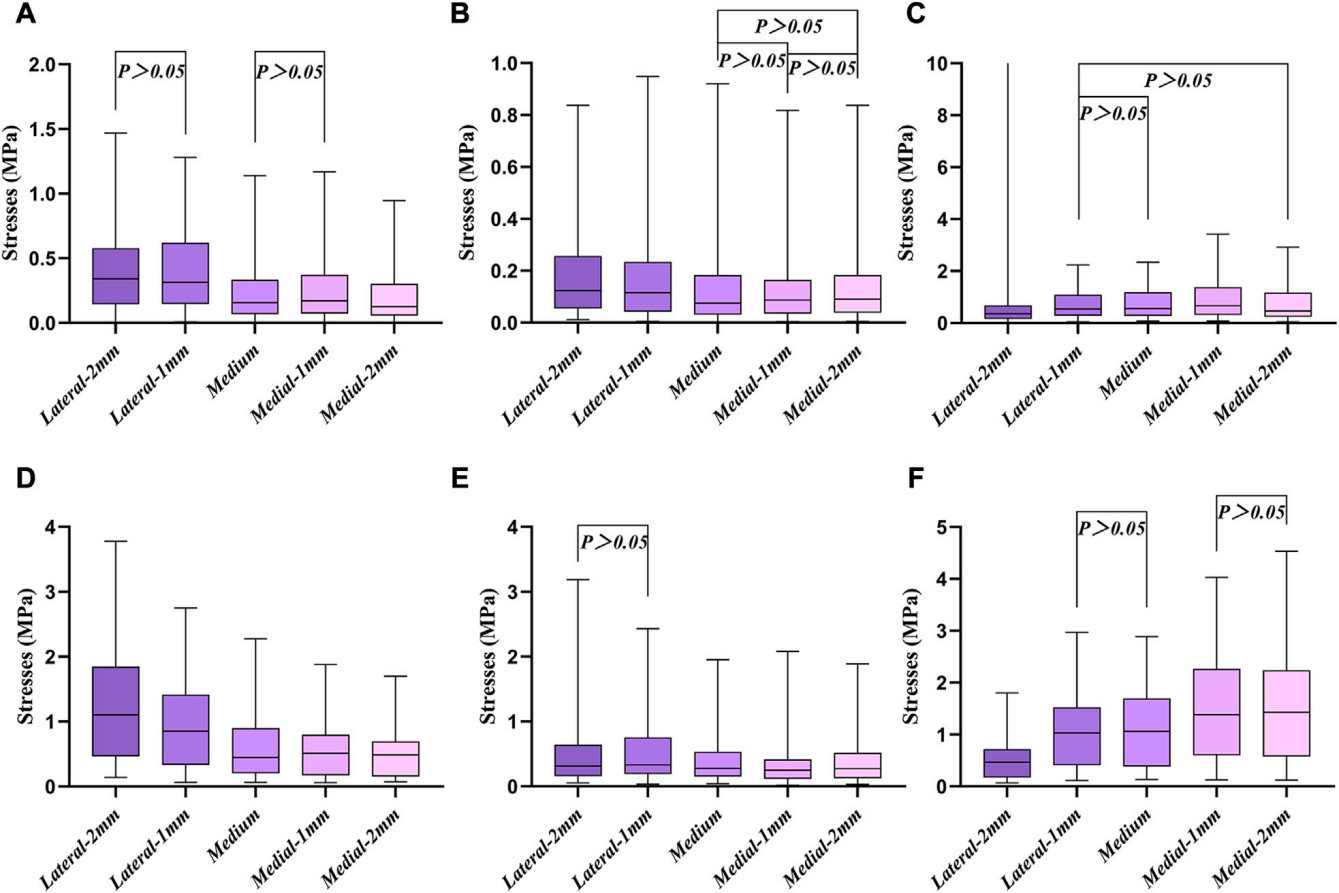
Box diagram of the first six different regions. **(A–F)** Six different regions of stress, corresponding to Figure 5, respectively.

The proximal tibia was divided into seven regions to analyze the stress distribution (Figure 5). All cell points in each region were extracted separately and the data was analyzed using SPSS software. Since the data does not obey a normal distribution, the results were analyzed using the Kruskal–Wallis test. According to the stress distribution in the first six regions, the data statistics were obtained as shown in Figure 6. Significant differences were found between the groups except for those labeled *p* > 0.05. The largest median of 1.43 MPa occurred in the case of a 2 mm medial misalignment in the F region, while the smallest median of 0.075 MPa was seen in the B region where the prosthesis was in the middle position. It can be seen that the red part of the high stress was concentrated in the lower part of the proximal tibia which was the G region.

**FIGURE 7:**
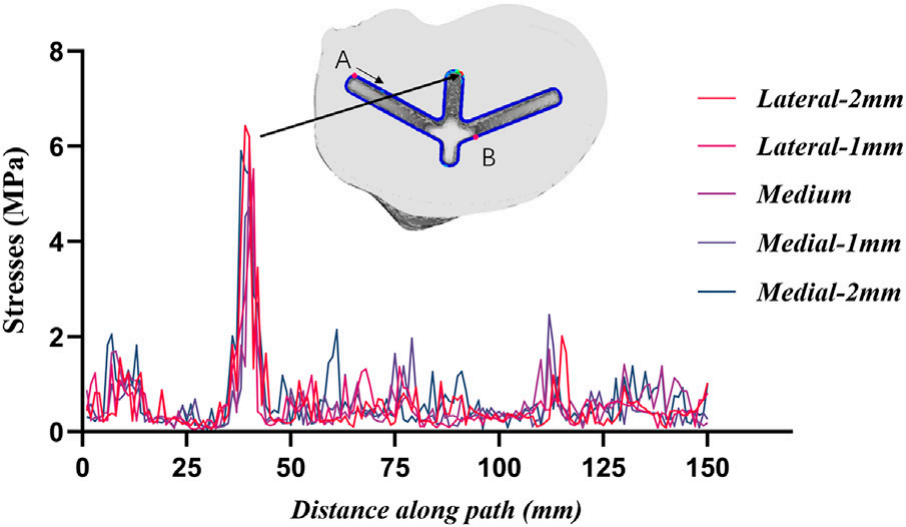
Stresses along the border of the stem cavity. Point A is the starting point and point B is the 100 mm position.

A representative evaluation path was defined for qualitative and quantitative comparison among the different positions of stresses at the region of the tibia plateau. Figure 7 shows stresses along the path defined at the stem cavity border. The major differences along this path pertain to the extreme values at the distance of 25–50 mm. The maximum stress along the defined path occurred in the lateral-2 mm group; the maximum stress in this case was 6.43 MPa. In the same location, the least stress model represents the medium group, with the maximum value of 1.38 MPa.

### 3.2 Micromotion

**FIGURE 8:**
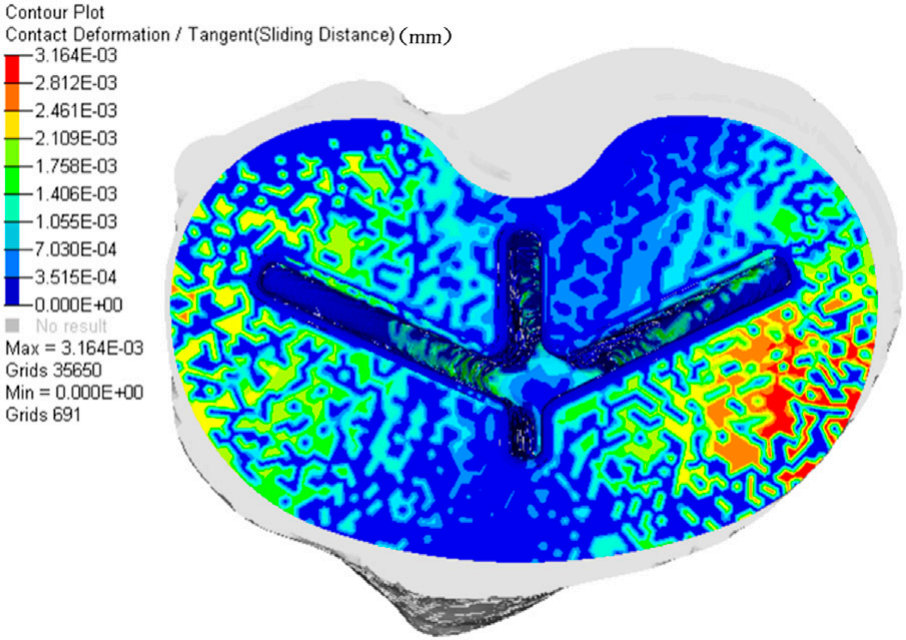
Micromotion cloud map when the platform is misaligned 1 mm to the lateral side.

**FIGURE 9:**
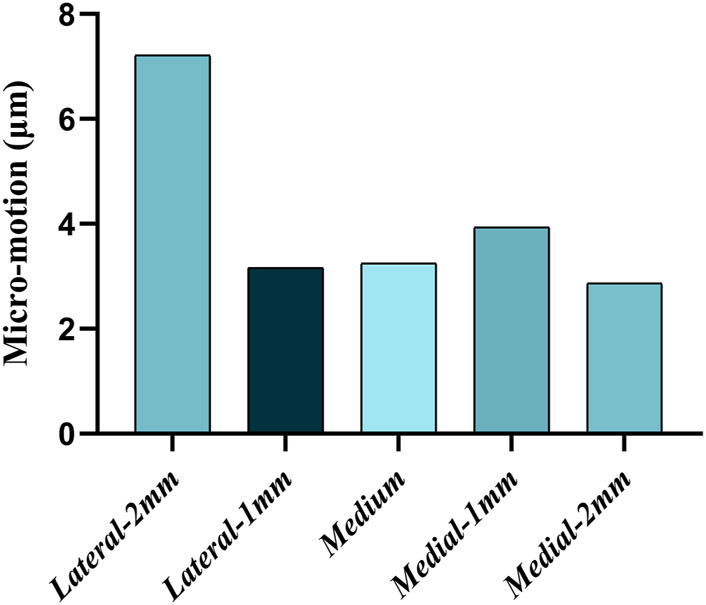
Micromotion at different misalignment positions of the platform.

**FIGURE 10:**
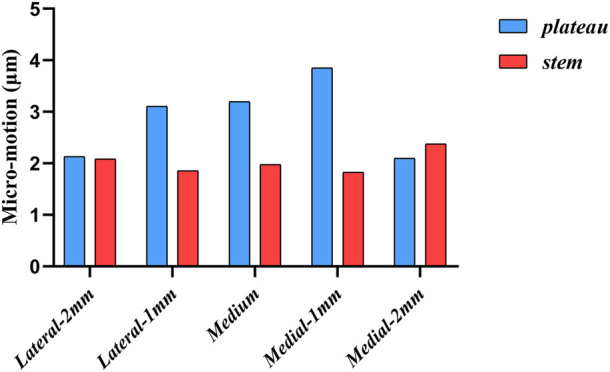
Micromotion at different misalignment positions of the plateau and stem.

Figure 8 shows the micromotion clouds of one of the groups and Figure 9 demonstrates the micromotion of the five sets of platforms for different misalignment situations. When the platform was misaligned to the lateral side by 2 mm, its micromotion of 7.215 μm was significantly larger than the remaining four groups. Additionally, while the platform was misaligned 2 mm medially, its micromotion of 2.869 μm was the smallest among the five groups. The middle three groups of micromotions are almost half of the first group, respectively. Figure 10 shows the micromotion at different misalignment positions of the plateau and stem. The largest micromotion of 3.854 um occurred on the plateau when the platform was misaligned 1 mm medially.

## 4 Discussion

Addressing the problems caused by the wear of prostheses in TKA remains a challenge for orthopedic surgeons. In previous studies, varus-valgus, anterior-posterior tilt, and internal and external rotation misalignment of the prosthesis were included (Fraser et al., 2015). However, no studies have been conducted to investigate the medial-lateral translational misalignment of the prosthesis.

FEA is an effective tool to evaluate the mechanical properties of prostheses and bone. The accuracy of the analysis depends on the veracity of the model. In this study, a non-homogeneous 3D tibia model was used, which means that it will be closer to real human bone than a homogeneous bone model, making the results of the study more realistic (Venalainen et al., 2016; Ün and Çalık, 2016). According to Ruggiero et al. (2019) we can learn that the quality of the hexahedral mesh is better than that of the tetrahedral one, especially in the dynamic condition. But based on the static conditions of our study and the fact that we draw the relatively small mesh, the accuracy is guaranteed even if we use the tetrahedral mesh in this study.

The comparison of the five groups of maximum stresses shows that the maximum stress was 47.29 MPa, which is less than the ultimate stress of 80 MPa in cortical bone (Maslov et al., 2021). This indicates that translational misalignments of 2 mm or less do not result in fractures. As can be seen in Figure 6, the stresses in the A and D regions were high when the platform prosthesis was misaligned to the lateral side, which was in line with our expectations. A similar pattern could be seen in regions C and F, when the tibial plateau prosthesis was transferred medially. Even with these stress concentrations, the stresses were not sufficient to cause significant effects on the proximal tibia (Maslov et al., 2021). Figure 7 demonstrates that, at the stem cavity border, the stresses are concentrated at the rear. Figure 9 shows that the largest micromovement was 7.215 μm, which was much smaller than 28 μm, showing that all five groups could have good bone growth in the future (Pilliar et al., 1986; Jasty et al., 1997). From Figure 10 it can be seen that the plateau presents more micromotion than the stem.

In summary, there is no significant effect on the proximal tibia whether the tibial plateau prosthesis is misaligned medially or laterally by 2 mm or 1 mm. However, according to the current study, the tibial plateau prosthesis should be properly aligned on the tibial surface. A prosthesis overlapping the bony surface will have a negative impact on the surrounding soft tissues, such as the medial and lateral collateral ligaments, and especially upon the popliteal tendon (Bonnin et al., 2017). Dowson and Jin, (1986) showed that in synovial joints, micro-elastohydrodynamic action largely smooths the initial roughness of the cartilage surface, which was then considered a form of lubrication responsible for the significant tribological properties of synovial joints. Later, Ruggiero, (2020) reviewed several theories on the natural synovial lubrication phenomenon in human joints that have been proposed over the years. These indicated that the synovial soft tissue was also closely related to friction. In order to achieve basic stability during the postoperative period without the formation of an alignment, the resistance to movement between the bone and the implant is optimized by increasing the friction at the interface. This is necessary because excessive relative movements can inhibit bone growth due to wear and tear of the bone and formation of fibrous tissue at the implant interface, which can lead to loosening and pain (de Vries et al., 2022). Therefore, oversizing or malposition should be avoided when installing a tibial plateau prosthesis during TKA.

This study has some limitations. First, we applied the stress under static conditions, without introducing dynamic factors such as squatting or walking. And no additional loading conditions such as maximum force in the gait cycle were added in this study. Second, we did not consider the impact on the muscles and ligaments. Third, no synovial lubrication phenomena were considered in this study. Apart from these, Affatato et al. (2019) used *in-vitro* experimental investigation in the study of femoral prosthesis roughness. Such *in-vitro* experiments were not used in our study. In future studies, if the above details are included in the experiments, the results obtained may be more appropriate to clinical settings.

## 5 Conclusion

FEA of stress distribution and micromotion results showed that misalignment of the tibial plateau prosthesis by 2 or 1 mm medially or laterally during TKA did not significantly negatively affect the stress upon the proximal tibia. However, to prevent postoperative pain and stiffness due to impingement on the surrounding soft tissues and ligaments, it is important to consider and design the most appropriate prosthesis and ensure proper positioning for different patients.

## Data Availability

The raw data supporting the conclusion of this article will be made available by the authors, without undue reservation.
